# The Fracture Callus Is Formed by Progenitors of Different Skeletal Origins in a Site‐Specific Manner

**DOI:** 10.1002/jbm4.10193

**Published:** 2019-05-04

**Authors:** Yongmei Wang, Ling Chen, Misun Kang, Lin Ling, Faming Tian, Sun Hee Won‐Kim, Sunita Ho, Daniel D. Bikle

**Affiliations:** ^1^ Endocrine Unit, University of California San Francisco and Veterans Affairs Medical Center San Francisco CA USA; ^2^ Bioengineering & Biomaterials Micro‐CT and Imaging Facility University of California, San Francisco San Francisco CA USA

**Keywords:** FRACTURE REPAIR, BONE FORMATION, LINEAGE TRACING, PROGENITOR

## Abstract

We evaluated repair following a mid‐diaphyseal fracture of the tibia in 3‐month‐old mice. We observed differences in the repair process at three different sites of the callus. Site 1: bone developing from the outer layer of the periosteum of the cortex; site 2: bone developing within the bridge/central region of the fracture; and site 3: bone developing within the marrow of the ends of broken bones. We characterized these sites by correlating datasets from X‐ray CT and histology. Correlated data demonstrated the involvement of different cells and different rates of mineralization. The origin of the progenitors and mechanism of progenitor differentiation involved at these sites was then evaluated using lineage tracing of cells expressing Prx1 and Col.2. The Prx1 progeny contributed to intramembranous bone formation (IBF) at site 1 and endochondral bone formation (EndoBF) at site 2 but not to intramedullary bone formation (IMBF) at site 3. IBF at site 1 was confirmed without a chondrocyte intermediate unlike EndoBF at site 2. Additionally, the presence of Col.2 progeny contributed to EndoBF in site 2 and IMBF in site 3 but not to IBF in site 1. However, the Col.2 progeny in IMBF in site 3 appeared to come from Col.2‐expressing osteocytes originating in the cortices of the ends of the fractured bone. In conclusion we have identified three sites of bone fracture repair that differ in their origin of cells and their mechanisms of bone formation. © 2019 The Authors JBMR Plus published by Wiley Periodicals, Inc. on behalf of American Society for Bone and Mineral Research.

## Introduction

Fractures are one of the most frequent injuries of the musculoskeletal system. Approximately 16 million bone fractures occur in the United States each year. Ten percent to 20% of fractures are complicated by delayed union or persistent nonunion, resulting in prolonged disability, impairing the quality of life and inflating healthcare costs.^1^, ^2^ A more mechanistic understanding of fracture repair may prevent delayed/nonunion and improve the effectiveness of fracture treatment.

Fracture repair is a complex process directed at restoring the original structures and biomechanical functions of bone. Many biological events, including inflammation, signaling, gene expression, cellular proliferation and differentiation, osteogenesis, chondrogenesis, angiogenesis, and remodeling occur during fracture repair.^3^ Traditionally, the processes have been best described for murine fracture repair in four overlapping stages: (i) hematoma formation and initiation of inflammation; (ii) soft callus formation; (iii) hard callus formation and initiation of bony union; and (iv) bone remodeling.^4^, ^5^, ^6^ Fracture repair recapitulates the process of embryonic development with the coordinated participation of a number of cell types.^7^, ^8^, ^9^ Several potential sources of skeletal stem cells/progenitors have been reported including endosteum,^10^, ^11^ periosteum,^12^, ^13^, ^14^, ^15^ bone marrow,^16^, ^17^and adjacent soft tissues.^18^ Among these sources, periosteum and bone marrow have been identified as main sources of progenitors for fracture repair.^19^ Similar to embryonic bone development, fracture repair involves a combination of intramembranous bone formation (IBF), in which progenitor cells directly differentiate into osteoblasts, and endochondral bone formation (EndoBF), in which the progenitors differentiate into chondrocytes, produce cartilage matrix (soft callus), which is eventually replaced by bone.^8^, ^20^, ^21^, ^22^ The environment at the site of the injury, for example the presence of hypoxia and the mechanical instability of the bone ends, affects the fate decision of the progenitor cells to enter either IBF or EndoBF. Stabilized fractures with normal oxygen tension heal with IBF, whereas nonstabilized fractures and hypoxia induce EndoBF.^23^ Despite the well‐characterized phases (soft tissue converting into a mineralized hard tissue) of fracture repair, the spatial temporal pattern of bone formation contributed by progenitors and resulting in fracture repair remains unclear. Determining the spatial pattern of bone formation and the origin and fate of the progenitor cells contributing to site‐specific development of the callus will provide direct and effective pathways that dictate fracture repair.

To address these issues, in the current study we generated spatial maps of the fracture callus identifying three different bone formation sites, then evaluated the structure, mineral density, morphology, and ultrastructure of bone forming at these sites during fracture repair. Moreover, we determined the contribution and fate of the progenitor cells at these bone forming sites by lineage tracing.

## Materials and Methods

### 2.1 Experimental animals and genotype

Tamoxifen regulated Prx1 GFP (tamprx1GFP) cre (a gift from Dr. Shunichi Murakami)^24^ or tamoxifen type IIa collagen (tamcol2) cre (a gift from Dr. Susan Mackem)^25^ were crossed with mice carrying the ROSA tomato transgene (tdT) (The Jackson Laboratory, Bar Harbor, ME, USA) to generate ^tamprx1GFP^Rosa tdTomato or ^tamcol2^Rosa tdTomato mice. All mice (five in each cage) were housed in a barrier facility with a 12‐hour light‐dark cycle, and maintained on standard chow. Mice were anesthetized with approved anesthetics (isoflurane) before procedures. For euthanasia, animals were exposed to isoflurane before cervical dislocation. Skeletons from male and their control littermates were analyzed. All animal studies were approved by the Animal Use Committee of the San Francisco Veterans Affairs Medical Center where the animals were raised and studied.

### 2.2 Genotyping

Genomic DNA was extracted from tail snips of the mice using REDTaq ReadyMix^™^ PCR Reaction Mix (Sigma‐Aldrich, St. Louis, MO, USA) following the manufacturer's instructions. PCR analyses of the DNA were performed to detect cres and tdT alleles (The Jackson Laboratory) using corresponding primer sets as described previously.^24^, ^25^

### 2.3 Nonstabilized fracture model

A closed tibia fracture was created by three‐point bending using Bose Electroforce 3200 mechanical instrument (Eden Prairie, MN, USA) as described.^26^, ^27^ Briefly, 3‐month‐old mice were anesthetized with 1% to 3% isoflurane. The right hind limb was placed on the lateral side, and an impactor was placed against the skin at the midpoint of the medial side of the lower leg. Then a preload of 1.0 N was applied before reaching a fracture impact at a constant speed of 0.2 mm/s. The fracture site occurred in the upper‐middle portion of the right diaphysis. Mice received analgesics after fracture and were returned to their cages and allowed to ambulate freely after awakening. Fractured and normal tibias were harvested for analysis at 10 and 28 days postfracture (*n* = 3 in each group).

### 2.4 X‐ray micro‐CT, scanning transmission electron microscopy, and energy dispersive X‐ray spectroscopy on fractured specimens

Each specimen was scanned under wet conditions (50% ethanol) using an X‐ray micro‐CT (µCT) unit (MicroXCT‐200; Carl ZEISS Microscopy, Pleasanton, CA, USA, at UCSF School of Dentistry, San Francisco, CA, USA). Scans were performed in absorption mode at 10 ×  magnification, peak voltage of 30 kVp, power at 8 W, current at 200 μA, and exposure time of 10 seconds. All acquisitions and image calculations were performed via XM Controller, Version 7.0.2817 and AVIZO data analysis software.

Following scanning of the specimen by X‐ray CT, it was infiltrated with LR‐white resin (Electron Microscopy Sciences, Hatfield, PA, USA). The infiltrated specimen was kept in a gelatin capsule (Electron Microscopy Sciences) and polymerized for 2 days at 60°C. Ninety‐nanometer‐thick (90‐nm‐thick) tissue sections from the fractured site were cut with an ultramicrotome (Reichert Ultracut E; Leica Microsystems, Inc., Buffalo Grove, IL, USA). The ultrasections were collected on formvar/carbon‐coated Ni grids (Electron Microscopy Sciences) for imaging using scanning transmission electron microscopy (STEM) (Sigma VP500; Carl Zeiss Microscopy) at 5 keV. The same sections were used to perform energy dispersive X‐ray spectroscopy (EDX) (Bruker AXS, Madison, WI, USA). Calcium (Ca) and phosphorus (P) elemental maps were collected. The ultrastructure as visualized by STEM was correlated with elemental maps of Ca and P as obtained using the EDX.

### 2.5 Histology

Tibias were fixed with 4% paraformaldehyde in PBS (4% PFA/PBS) overnight at 4°C and decalcified in 10% EDTA. To determine the tdT or GFP expression, decalcified bones were put into 30% sucrose overnight then embedded in O.C.T., and cut into 10‐µm sections. The sections were counterstained with DAPI and evaluated by fluorescence microscopy. For other histologic measurements, decalcified bones were embedded in O.C.T, and cut into 5‐µm sections. The sections were stained by H&E following standard procedures or subjected to immunohistochemistry for Sox2, osteocalcin, and dentin matrix protein‐1 (DMP‐1) expression with the appropriate antibodies at 1:200 dilution (Abcam, Cambridge, MA, USA) and counterstained with DAPI.

## Results

### 3.1 The three sites of bone fracture repair

**Figure 1:**
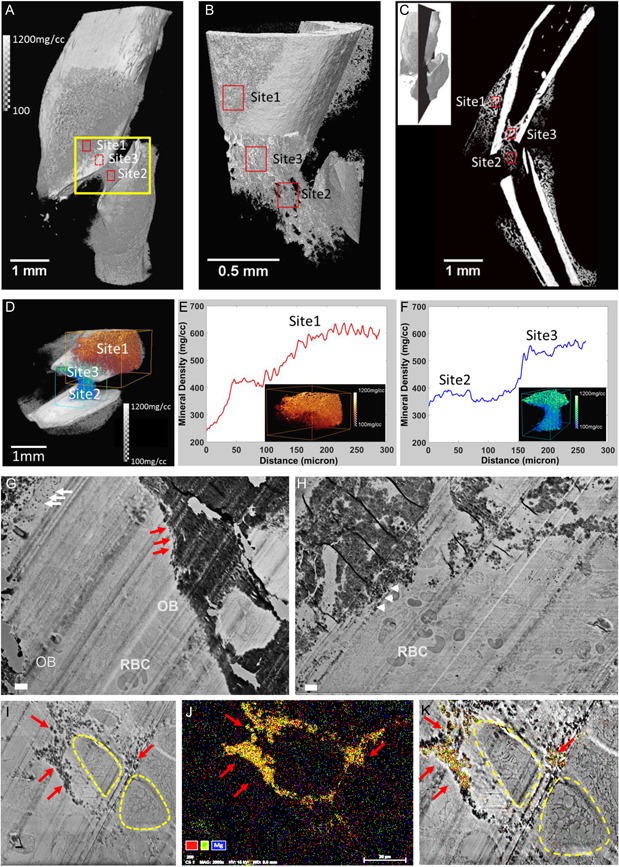
(A–F) Site‐specific regions of bone formation during fracture repair. (*A*) Volume‐rendered X‐ray CT image (magnification 4 × ) illustrates the three sites of interest (sites 1 to3). Grayscale values indicate bone mineral densities in mg/cm^3^. (*B*) Fractured area (the rectangular yellow box, *A*) is shown at magnification 20 × . The outer layer of the cortical bone was digitally peeled‐off to reveal site 3, the intramedullary site. (*C*) Virtual section illustrates all three sites at magnification 4 × . Inset indicates the location of the virtual section in volume rendered image. (*D*) Mineral density gradients across sites of interest at the fractured region. Red box: site 1 (*E*) and blue box: sites 2 and 3 (*F*) indicate average mineral density gradients (see color bars) over volumes of mineralizing callus. Note: see Supporting Movie 1. (*G*–*K*) Ultrastructure of mineralizing callus. SEM scan performed at day 10 postfracture shows mineralization nodules (white arrows) and mineralization front (red arrows), osteoblasts (OB), and red blood cells (RBC) at site 1 (*G*) and 3 (*H*). At site 2 (*I*–*K*), hypertrophic chondrocytes (HC, yellow dotted cycles) and mineralized matrix (*I*, cluster of black dots, indicated by arrows) around HCs were observed. EDX mapping revealed the mineralized matrix surrounding one of the HC (*J*, yellow, overlap with red and green): calcium (red) and phosphate (green). (*K*) overlap image showed the one HC surrounded by mineralizing matrix containing calcium and phosphate. Scale bars = 2 µm.

**Figure 2:**
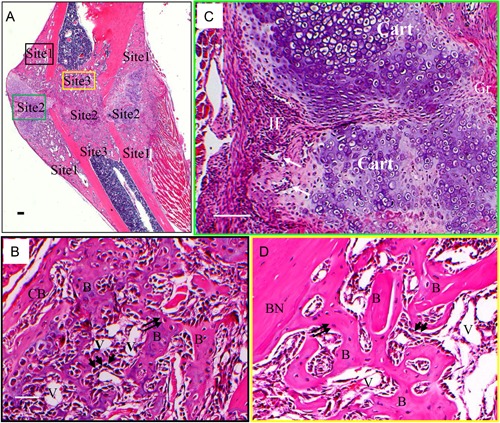
Morphology of the three bone formation sites of the mineralizing callus. (*A*) H&E staining indicates 3 bone formation sites. (*B*–*D*) High magnification of site 1 (*B*, black box in *A*), site 2 (*C*, green box in *A*), and site 3 (*D*, orange box in *A*). Arrows in *C*: vessels. Arrowheads in *B* and *D*: osteoblasts. Double arrows in *B* and *D*: osteocytes. Magnification 2.5 ×  in *A*, 5 ×  in *C*, scale bars = 100 µm; magnification 10 ×  in *B* and *D*, scale bars = 50 µm. B =  newly formed bone; CB =  newly formed cortical bone; V =  vessel; Cart =  cartilage; Gr =  granulated tissue. BN =  fractured bone (bone end).

Using X‐ray CT (Fig. 1) and histology (Fig. 2) we observed differences in mineral densities, and in the repair process at three different sites of the callus at day 10 postfracture—site 1: IBF developing from the outer layer of the periosteum of the cortex proximal or distal from the injury; site 2: EndoBF developing within the central bridge region of the fracture; and site 3: intramedullary bone formation (IMBF) developing within the marrow of the ends of broken pieces of the long bones. As determined by X‐ray CT, substantial differences in mineral density were observed at these three sites. Figure 1 *A*, *B* show the 3D structure of the fractured region, with a grayscale showing the differences in mineral density at the three sites. Fig. 1 *C* also contains a 2D slice through the fracture to illustrate the intramedullary location of site 3. Fig. 1 *D*–*F* show the change in mineral densities. At site 1 (red box in Fig. 1 *D*) the mineral density gradient varied from 250 mg/cm^3^ to 600 mg/cm^3^ with the lowest densities closest to the fracture site (see average density profile, Fig. 1 *E*), while at sites 2 and 3 (blue and green boxes, respectively, in Fig. 1 *D*), a gradient was observed from site 2 around 350 mg/cm^3^ to site 3 around 500 to 550 mg/cm^3^ (Fig. 1 *F*). The average mineral density profiles of these respective regions were site 1 >  site 3 >  site 2. Supporting Movie 1 provides a 360‐degree 3D look of the assignation of the three sites. The ultrastructure of these different sites was determined by SEM. At site 1, close to the fracture site and near the periosteum, active mineralization was observed as evidenced by mineralized nodules and adjacent osteoblasts (OBs) (Fig. 1 *G*). The presence of red blood cells (RBCs) in this image indicates marrow formation and normal oxygen tension within site 1. Similar to site 1, mineralized nodules were observed at site 3 of IMBF again with adjacent osteoblasts and RBCs marking the marrow (Fig. 1 *H*). On the other hand, in site 2 hypertrophic chondrocytes are found, some of which are involved in mineralizing the matrix (Fig. 1 *I*–*K*). The mineralized nodules containing calcium (Ca) and phosphate (P) are clearly revealed by EDX mapping (Fig. 1 *I*–*K*). Fig. 1K shows the overlay between the SEM image in Fig. 1I showing chondrocytes and the EDX (Fig. 1 *J*) imaging showing that only one of these chondrocytes is involved in the mineralization process. This is consistent with our understanding of EndoBF.

The histology of the three bone formation sites was evaluated by H&E staining (Fig. 2). At site 1 (Fig. 2 *A*, *B*), woven bone was formed. Osteoblasts located among the newly formed woven bone surface and large hypertrophic osteocytes were observed inside newly formed bone. Blood vessels were observed in the marrow spaces of the woven bone. This process formed the cortical shell of the callus. Neither chondrocytes nor cartilage were observed at site 1, indicating no EndoBF at this site. At site 2 (Fig. 2 *A*, *C*), at the gap between the fractured ends of the bone, the periosteum thickened in response to the fracture, with invasion into the gap area (invasion front, Fig. 2 *C*) accompanied by blood vessels (Fig. 2 *C*, arrows). Cartilage formed around the invasion front (Fig. 2 *C*, cart) with chondrocytes at various stages of differentiation. Immature chondrocytes were located right next to the invasion front, whereas mature chondrocytes were located distal to the invasion front, suggesting that the chondrocytes differentiated from the cells in the invasion front. Supporting this argument are the observed gradients in mineral densities. The lower mineral density at the invasion front gradually transitioning into higher mineral density is indicative of immature chondrocytes transforming into mineral‐producing chondrocytes.^28^ At site 3 (Fig. 2A, *D*), in the medullary areas within the fractured ends of the bone, trabecular bone forms, with blood vessels and marrow similar to that of metaphyseal bone. No cartilage or chondrocytes were observed, indicating no EndoBF at this site. The histologic appearance of these three sites confirms the observations from X‐ray CT regarding the differences in morphology shown in Fig. 1.

### 3.2 Contribution of Prx1‐positive osteochondroprogenitors during fracture repair

**Figure 3:**
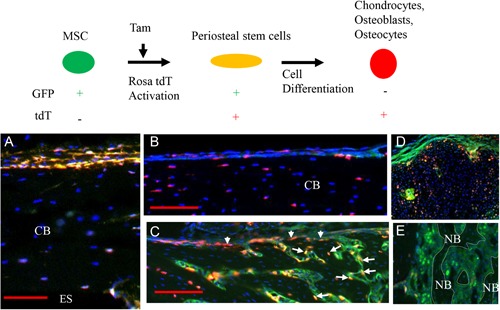
Fate of Prx1‐expressing cells during fracture repair. The cartoon in the upper panel illustrates the color changes in response to the administration of tamoxifen. Green dots mark cells still expressing tamprx1GFP. Red dots mark cells in which ^tamprx1GFP^Rosa tdTomato has been activated by tamoxifen but no longer express tamprx1GFP. Yellow dots mark cells with both green and red labels indicating continued expression of activated tamprx1GFP. As shown in the lower panel, tamoxifen was given 1 day before fracture of mice expressing ^tamprx1GFP^Rosa tdTomato. Following tamoxifen administration but before fracture (day 1) yellow fluorescence appears only in the periosteal surface (*A*), but not in the ES or osteocytes of CB. At day 10, tdTomato‐labeled cells (red) appeared in the periosteum and osteocytes of CB distant to the fracture site (*B*). At day 10 postfracture, dual‐labeled cells were observed in the periosteum (yellow, arrowheads) and newly formed bone surface (yellow, arrows) in site 1 (*C*), in the cartilage in site 2 (*D*), but not in site 3 (*E*). Nuclei were counterstained by DAPI (blue dots). NB indicated by dotted lines in *E*. Magnification 10 ×  in *A*–*E*, scale bars = 100 µm. ES =  endosteal surface; CB =  cortical bone; NB =  newly formed bone.

Prx1 is a marker of the mesenchymal lineage in developing limbs. Prx1‐expressing cells within adult bones are found primarily in the periosteum and have been shown to participate in bone fracture repair.^24^ To determine the temporal involvement of Prx1‐expressing cells in the three fracture sites during fracture repair, tamoxifen (tam) was given 1 day before fracture to ^tamprx1GFP^Rosa tdTomato cre mice. Following tam administration but before fracture (day 0) only GFP (Prx1‐positive cells, green) and tdTomato (activated Prx1‐positive cells, red) dual‐labeled cells (yellow) appeared in the periosteum (Fig. 3 *A*), but not in the endosteum (ES) (Fig. 3 *A*). At day 10 postfracture (and 11 days post‐tam administration), tdTomato‐labeled cells (red) appeared in the periosteum and osteocytes embedded in the cortical bone distant to the fracture site (Fig. 3 *B*), but GFP‐labeled cells or dual‐labeled cells were no longer found. However, at the fracture site a large number of dual‐labeled cells as well as single‐labeled tdTomato cells were observed in the periosteum and newly formed bone in site 1(Fig. 3 *C*). Dual‐labeled cells also appeared in the cartilage in site 2 (Fig. 3 *D*), but no tdTomato‐labeled cells were observed in site 3 (Fig. 3 *E*). These data indicate that during fracture repair, Prx1‐expressing osteochondroprogenitors contribute to both IBF at site 1 and EndoBF at site 2, but do not contribute to IMBF at site 3.

### 3.3 Contribution of Col.2‐positive progenitors during bone development and fracture repair

**Figure 4:**
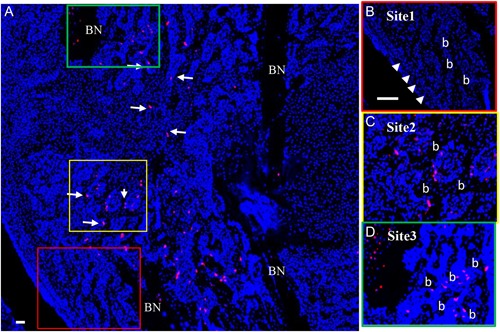
Fate of Col.2‐expressing cells during fracture repair. Tamoxifen was given 1 day before fracture to mice expressing ^tamcol2^Rosa tdTomato, and the callus imaged 10 days postfracture. Nuclei were counterstained with DAPI (blue dots). Labeled cells (red dots) appeared at the newly formed bone surface (b) in the bridge region (site 2) (arrows in *A*, *C* =  yellow box in *A*), cortical bone of the fractured bone ends (BN), and intramedullary area (site 3) (*A*, *D* =  green box in *A*), but not in the bone surface (*A*, *B* =  red box in *A*) formed from periosteum (arrows in *B*) in site 1. Magnification 5 ×  in *A*, scale bar = 100 µm; magnification 10 ×  in *B*–*D*, scale bar = 50 µm. b =  new formed bone; BN =  bone ends.

During bone development and fracture repair, Col.2‐positive progenitors, differentiating into chondrocytes, provide the initial stages of EndoBF. At least a fraction of these chondrocytes subsequently transdifferentiate into osteoblasts.^29^, ^30^, ^31^ To determine the cell fate of Col.2‐positive cells during bone development, tam was given at embryonic day 15.5 to ^tamcol2^Rosa tdTomato mice, and the labeled cells were evaluated at postnatal day 1 (P1) and day 14 (P14) after birth. As shown in Supporting Fig. 1, at day 1, tdTomato‐expressing cells appeared in all zones of the growth plate and metaphysis with particular concentration just inside the perichondrium and articular surface (Supporting Fig. 1 *A*). At day 14, most of the tdTomato‐positive cells appeared in the bone surface of the primary (metaphysis) and secondary (epiphysis) ossification centers (Supporting Fig. 1*B*). Unexpectedly, tdTomato‐labeled osteocytes also appeared in the cortical bone of the mid‐diaphysis (Fig. 4, Supporting Fig. 1*C*). As controls for our fracture studies, we also analyzed the nonfractured tibia in the 3‐month‐old mice for the expression of Col.2. Consistent with the studies during bone development, the progeny of ^tamcol2^Rosa tdTomato mice following tam administration were observed in the growth plate and at the bone surface within the primary and secondary ossification centers (data not shown) as well as in the cortical bone of the mid‐diaphysis (Supporting Fig. 1*C*). No tdTomato‐positive cells were observed in the perichondrium (Supporting Fig. 1 *A*, *B*), periosteum, endosteum, or bone marrow (Supporting Fig. 1*C*) at either age. To determine the contribution of Col.2‐positive progenitors during fracture healing, tam was given 1 day before fracture to mice expressing ^tamcol2^Rosa tdTomato, and the location of tdTomato‐positive descendants was determined 10 days after fracture (Fig. 4). At this time point ^tamcol2^Rosa tdTomato‐positive cells appeared on the bone surfaces in site 2 (Fig. 4 *A*, *C*). However, in contrast to ^tamprx1^Rosa tdTomato‐labeled progeny,^tamcol2^Rosa tdTomato‐labeled cells were also observed in the newly formed bone at site 3 (Fig. 4 *A*, *D*), but not in site 1, the periosteum, or endosteum (Fig. 4 *A*, *B*). Moreover, in the cortical bone of the fractured bone ends, a large number of osteocytes were labeled (Fig. 4 *A*, *D*) as was seen in the nonfractured tibia of the 14‐day‐old mouse (Supporting Fig. 1 *C*). These data indicate that Col.2‐positive progenitors contribute to EndoBF at site 2 and IMBF at site 3, but do not contribute to IBF at site 1. Moreover, these data strongly suggest that Col.2‐expressing osteocytes in the cortical bone are the source of osteoblasts for IMBF during fracture repair.

### 3.4 Characterization of Col.2‐derived cells

**Figure 5:**
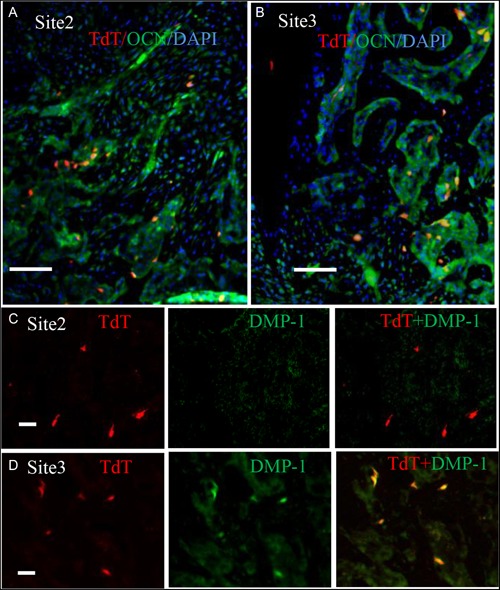
^tamcol2^Rosa tdTomato‐positive cells co‐expressed osteocalcin (sites 2 and 3) or DMP‐1 (site 3). At day 10 postfracture, immunohistochemistry using osteocalcin antibody (green) showed that ^tamcol2^Rosa tdTomato‐positive cells (red) in site 2 (*A*) and 3(*B*) also expressed osteocalcin (yellow or orange, red+green). Nuclei were counterstained DAPI (blue). On the other hand, immunohistochemistry identified that in site 3 (*D*) ^tamcol2^Rosa tdTomato‐positive cells (red, right) co‐expressed (yellow or orange) DMP‐1 (green), but in site 2, no expression of DMP‐1 was observed in the ^tamcol2^Rosa tdTomato‐positive cells (*C*). Magnification 10 ×  in all, scale bars = 50 µm.

To further identify Col.2‐expressing cells in sites 2 and 3, we performed immunohistochemistry for Col.2. Recent studies showed that a subset of Col.2‐positive progenitors transdifferentiate into osteoblasts during fracture repair,^29^, ^30^ and that these cell types may include osteocytes.^32^ We first used an osteocalcin antibody to determine that the ^tamcol2^Rosa tdTomato‐positive cells in sites 2 and 3 were osteoblasts. As shown in Fig. 5, osteocalcin expression colocalized with tdTomato in many of the cells in site 2 (Fig. 5 *A*) and site 3 (Fig. 5 *B*). These results support the concept that a subset of Col.2‐positive progenitors transdifferentiate into osteoblasts, contributing to both EndoBF at site 2 and IMBF at site 3. As noted earlier, chondrocytes were not found during IMBF, supporting the concept that the origin of the cells forming bone in site 3 originate from the adjacent cortical bone, namely the Col.2‐expressing osteocytes. To further study this possibility we used a DMP‐1 antibody to further evaluate whether ^tamcol2^Rosa tdTomato‐positive cells in site 3 are derived from osteocytes in the cortices of the fractured bone. As shown in Fig. 5C, *D*, DMP‐1 (green) colocalized with the ^tamcol2^Rosa tdTomato‐positive progeny (red) at site 3 (Fig. 5 *D*), but DMP‐1 expression was not observed in the ^tamcol2^Rosa tdTomato‐positive cells at site 2 (Fig. 5 *C*). These observations further support the concept that Col.2‐expressing osteocytes from the cortex of the fractured bone are major contributors to IMBF, but not to bone formation at the other sites.

### 3.5 Characterization of Col.2‐positive osteocytes

**Figure 6:**
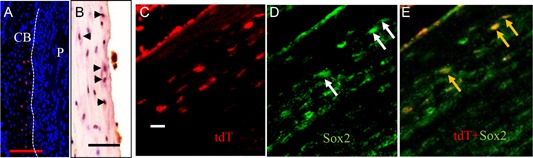
^tamcol2^Rosa tdTomato‐positive cells in cortical bone have reprogramming potential. At day 10 postfracture, ^tamcol2^Rosa tdTomato‐positive cells appeared in the normal mid‐diaphysis of the CB (*A*, red dots) or CB ends in the callus. Immunohistochemistry confirmed that Col.2 (*B*, brown) was expressed in these cells. Furthermore, in the CB ends in the callus the ^tamcol2^Rosa tdTomato‐positive cells (*C*, red) co‐expressed (*E*, yellow or orange, arrows) Sox2 (*D*, green, arrows). Magnification 10 ×  in *A*–*E*, scale bars = 50 µm. CB =  cortical bone.

To verify that the ^tamcol2^Rosa tdTomato‐positive cells in the mid‐diaphysis of the cortical bone (CB, red dots) (Fig. 6 *A*) actually produce Col.2, we performed immunohistochemistry with an antibody to Col2a1, and demonstrated that Col.2a1 was expressed in these cells (Fig. 6B, brown). To determine whether these cells have the potential to reprogram, and thus contribute to bone formation during fracture repair, we evaluated their expression of Sox2, a marker for stem cell activation. As shown in Fig. 6 *C–E*, Sox2 (green) colocalized (yellow or orange) with the ^tamcol2^Rosa tdTomato‐labeled (red) osteocytes in the fractured bone cortices.

## Discussion

In the current study of fracture repair we demonstrated bone formation at three different sites involving different processes. Mesenchymal osteochondroprogenitors from the periosteum contribute to IBF adjacent to the fracture site to help form the outer bony surface of the callus without an intermediate chondrocyte stage (site 1). These cells also contribute to the EndoBF occurring in the gap between the fractured bone ends that helps bridge this gap (site 2). Finally, and most surprisingly, osteocytes within the cortices of the fractured bone appear to be the source of bone formation within the intramedullary regions of the ends of the fractured bone (IMBF) that like IBF does not go through a chondrocyte stage despite the ability of these osteocytes to produce Col.2. These different characteristics of fracture healing at the three sites are summarized in Supporting Table 1.

We used histology and X‐ray CT to initially identify the three sites of bone fracture repair. Histologically, based on the pattern of bone formation, callus formation could be divided into three different bone formation sites based on the appearance of the cells involved and their location within the fracture site. X‐ray CT further distinguished the three sites by determining their spatial structure and mineral density. Scanning electron microscopy combined with EDX further confirmed the structure and specific elemental distribution in the three sites. We found that on average the gradient of mineralization from site 2 to site 3 was steeper than the gradient in site 1 from the region adjacent to the fracture to the region further from the fracture. These differences in gradients (abrupt from site 2 to site 3, and relatively gradual along site 1) are indicative of differences in mineralization rates at these three sites.

We then turned our attention to the origins and cell fates of the progenitor cells contributing to fracture repair at these three sites. Previous studies demonstrated that the periosteum is a primary source of progenitor cells contributing to fracture repair.^12^, ^13^, ^15^ A subset of cells in the periosteum express Prx1 and preferentially differentiate into osteogenic and/or chondrogenic lineages in vitro. These osteochondroprogenitor cells are major contributors to fracture repair.^24^, ^34^ Our data demonstrate that in response to injury, the Prx1‐expressing cells in the periosteum are activated and pursue two different fates. Prx1‐expressing cells in the periosteum adjacent to the fracture site differentiate directly into osteoblasts. They form woven bone at site 1 on the outside of the existing cortical bone through IBF, initiating formation of the hard callus. In contrast, the Prx1‐expressing cells located at the fracture site invade the fracture site, differentiating initially into chondrocytes, forming cartilage to bridge the fracture gap (site 2). Previous studies^29^, ^35^ demonstrated that in the inducible Prx1GFPCreErt model which we used for these studies, Prx1‐labeled cells were restricted to the periosteum, and were not found in the endosteum or bone marrow, the two potential sources of progenitors for bone formation in the medullary cavity (site 3).^12^ Consistent with these studies, no activated Prx1‐expressing cells were observed in site 3, indicating that these mesenchymal cells of the periosteum do not contribute to IMBF.

During EndoBF, Col.2 is expressed in proliferating chondrocytes and is generally used as a chondrocyte marker. In the current study, compared with the Prx1‐expressing cells, ^tamcol2^Rosa tdTomato‐positive cells have the following distinctive features. First, lineage tracing experiments demonstrated that during either embryonic or postnatal development, ^tamcol2^Rosa tdTomato‐positive cells did not appear in the periosteum—the main source of cells contributing to site 1. Moreover, ^tamcol2^Rosa tdTomato‐positive cells did not appear in the periosteum in response to injury, and did not contribute to the IBF in site 1 following fracture. On the other hand, ^tamcol2^Rosa tdTomato‐positive progeny contribute to EndoBF at site 2, presumably as progeny of the mesenchymal cells in the periosteum, appearing on the newly formed bone surfaces of site 2. On the bone surface, these cells co‐expressed the osteoblast marker osteocalcin, indicating that during fracture repair, a subset of ^tamcol2^Rosa tdTomato‐positive cells transdifferentiate into osteoblasts, contributing to EndoBF, confirming earlier observations.^29^, ^30^, ^31^ However, ^tamcol2^Rosa tdTomato‐positive progenitors also contributed to medullary bone formation at site 3 even though chondrocytes were not identified in this region. These cells co‐expressed the osteoblast marker osteocalcin as well as the osteocyte marker DMP‐1, indicating these cells have osteoblast and osteocyte features. Previous studies showed that osteoblasts in this region were differentiated from the endosteum/bone marrow,^12^ but our lineage tracing data indicated no ^tamcol2^Rosa tdTomato‐positive cells in the endosteum/bone marrow, either during bone development or fracture repair, suggesting that these cells originated from another source. To our surprise, osteocytes in the mid‐diaphysis in both nonfractured and fractured bones expressed Col.2. Osteocytes in the proximal or distal portions of the diaphysis do not. Whether a specific set of osteocytes retain this marker of their origin from chondrocytes during embryonic development, or regain the expression of Col.2 in the mature bone is not clear, nor is the reason that only the osteocytes in the mid‐shaft of the tibia express Col.2. In support of the chondrocyte origin of these osteocytes are the studies showing that hypertrophic chondrocytes can transdifferentiate into osteoblasts, which further differentiate into osteocytes embedded into the bone matrix.^29^, ^31^, ^35^, ^36^ Similarly, during the fracture repair, in response to the injury, Col.2‐expressing chondrocytes in the soft callus transdifferentiate into osteoblasts, then differentiate into osteocytes.^30^ Although osteocytes are thought to be terminally differentiated cells (as were hypertrophic chondrocytes), perhaps those cells expressing Col.2 have the potential to proliferate or differentiate into other cells when activated by fracture as previously suggested.^32^ Our data support this possibility, as we showed that ^tamcol2^Rosa tdTomato‐labeled osteocytes co‐express the stem cell marker Sox2, suggesting that in response of injury, these cells have the potential to reprogram to osteoprogenitors to contribute to fracture repair. Thus Col.2‐expressing osteocytes appear to be the source of osteoblasts for IMBF. Moreover, the ^tamcol2^Rosa tdTomato‐expressing cells at site 3 express DMP‐1, further indicating that these cells are of osteocyte origin, and that following fracture the ^tamcol2^Rosa tdTomato‐labeled osteocytes are reprogrammed, migrate out of the ends of the broken bone, and form the bone in the intramedullary site. Further studies are needed to confirm this hypothesis.

In summary, during fracture repair, Prx1‐expressing and Col.2‐expressing cells contribute to fracture repair at different sites by different mechanisms. Prx1‐expressing osteochondroprogenitors from the periosteum contribute to IBF and EndoBF, but not IMBF. A subset of Col.2 expressing chondrocytes transdifferentiates into osteoblasts and contributes to EndoBF at site 2, but not at site 1 or 3. At the ends of the fractured bone, Col.2‐expressing osteocytes appear to reprogram, differentiate into osteoblasts, and contribute to IMBF at site 3.

## Disclosures

All authors state that they have no conflicts of interest.

## Supporting information

Supporting Information

Supporting Information

Supporting Information

Supporting Information
