# Supplementary figures and images for: The Fracture Callus Is Formed by Progenitors of Different Skeletal Origins in a Site‐Specific Manner
Source: JBMR Plus. 2019 May 4;3(9):e10193. doi: 10.1002/jbm4.10193 (PMC6808225; doi:10.1002/jbm4.10193)

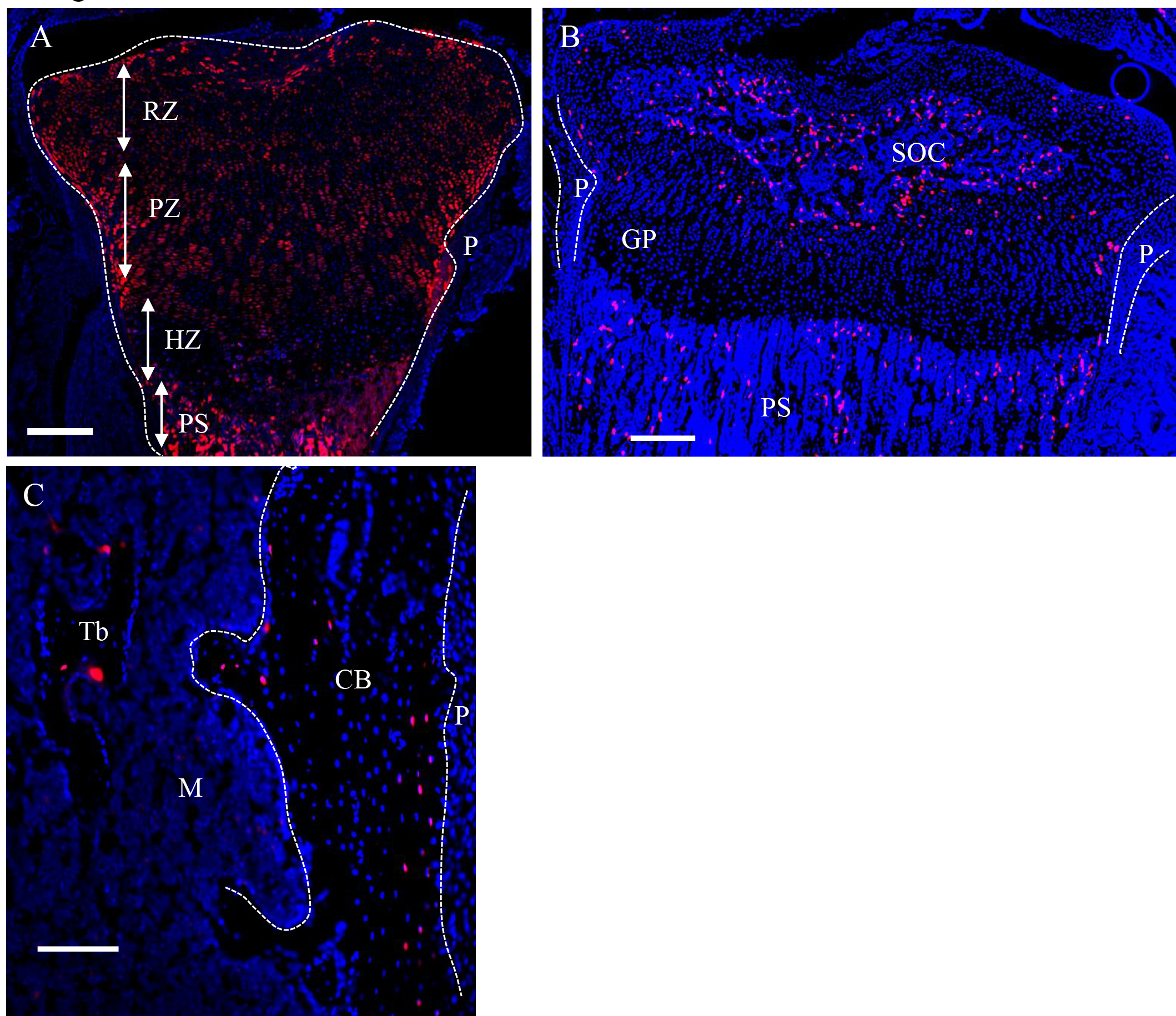

Supplement: Supplementary file 1 — Supporting Information [file JBM4-3-na-s001.tif]
