# Supplementary material for: The Fracture Callus Is Formed by Progenitors of Different Skeletal Origins in a Site‐Specific Manner
Source: JBMR Plus. 2019 May 4;3(9):e10193. doi: 10.1002/jbm4.10193 (PMC6808225; doi:10.1002/jbm4.10193)
Supplement: Supplementary file 3 — Supporting Information [file JBM4-3-na-s003.docx]

| Site | Location | Origin of progenitors | Bone formation process | Mineralization rate |
| --- | --- | --- | --- | --- |
| 1 | periosteum | periosteum | intramembranous | slow to rapid from fracture site |
| 2 | bridge | periosteum | endocortical | slow |
| 3 | medulla | cortical osteocytes | intramedullary | rapid |

Table1: Characteristics of the 3 sites of fracture repair
