# Supplementary material for: The Fracture Callus Is Formed by Progenitors of Different Skeletal Origins in a Site‐Specific Manner
Source: JBMR Plus. 2019 May 4;3(9):e10193. doi: 10.1002/jbm4.10193 (PMC6808225; doi:10.1002/jbm4.10193)
Supplement: Supplementary file 4 — Supporting Information [file JBM4-3-na-s004.docx]

**Supplemental Movie S1. showing the 3 sites of fracture repair.** The movie provides a 360-degree 3D look at the structure of the callus 10d after fracture. Newly formed membranous bone can be seen on the outside of the fractured bone cortices (site 1) and inside the medullary cavity close to the bone ends (site 3). In contrast, a radiolucent gap is seen between the fractured bone ends where endochondral bone formation is taking place (site 2).

**Figure S1. Cell fate of ^tamcol2^Rosa tdTomato expressing cells during bone development**. Tamoxifen was given at E15.5 to mice expressing ^tamcol2^Rosa tdTomato: at P0 (A) tdTomato labeled cells (red dots) appeared in the resting (RZ), proliferation (PZ) and hypertrophic (HZ) zones of the growth plate (GP), and primary spongiosa (PS). At P14 (B) a few of the labeled cells appear in the GP, but most of them had migrated to the metaphysis (PS) and epiphysis (secondary ossification center, SOC). C: Tamoxifen was given one day before fracture. tdTomato labeled cells (red dots) appeared in the mid-diaphysis and trabecular bone surface (Tb) of the non-fractured tibia at day 10 post fracture. No tdTomato labeled cells were observed in the bone marrow (M), periosteum (P) and endosteum (dotted line). Dotted lines indicate the boundary of GP (A&B) and CB (C). 5 X in A and B, bars = 100 µm, 10 X in C, bar = 50 µm.
